# Sarcome d'Ewing de localisation pétreuse

**DOI:** 10.11604/pamj.2015.22.260.8223

**Published:** 2015-11-19

**Authors:** Rim Lahiani, Madiha Mahfoudhi

**Affiliations:** 1 Service d'ORL, Hôpital Charles Nicolle, Tunis, Tunisie; 2 Service de Médecine Interne A, Hôpital Charles Nicolle, Tunis, Tunisie

**Keywords:** Sarcome d′Ewing, rocher, chimiothérapie, Ewing sarcoma, petrous bone, chimiotherapy

## Image en medicine

Le sarcome d'Ewing est une tumeur osseuse agressive, rapidement évolutive et métastatique. La localisation pétreuse est rare. Elle présente des difficultés diagnostiques et thérapeutiques. La prise en charge doit être multidisciplinaire. Le meilleur traitement reste chirurgical au prix de séquelles fonctionnelles et esthétiques. L'avènement de nouvelles molécules de chimiothérapie a amélioré le pronostic. Garçon âgé de 16 mois, sans antécédentsparticuliers a présenté une otorragie spontanée gauche. L'examen physique a révélé une formation charnue, saignante, non battante, comblant le conduit auditif externe gauche. Le patient n'avait pas de paralysie faciale ni d'adénopathies cervicales palpables. La TDM des rochersa révélé un processus tissulaire du conduit auditif externe gauche, étendu à l'oreille moyenne avec lyse du mur de la logette et de l'os temporal. L'IRM des rochers a montré le même processus avec envahissement de la parotide gauche, en hyposignal T1, se réhaussant après injection de gadolinium. L'examen anatomo-pathologique d'une biopsie osseuse a confirmé le diagnostic d'un sarcome d'Ewing. Le bilan d'extension n'a pas retrouvé de métastases. La chimiothérapie d'induction (6 cures de Vincristine, Ifosfamide, Doxorubicine et Etoposide) a permis une réduction tumorale de plus que 80%. Une intervention chirurgicale a consisté en une pétrectomie gauche, parotidectomie exo-faciale conservatrice et un curage triangulaire. L'examen histologique a confirmé un sarcome d'Ewing avec des limites de résection saines. Une chimiothérapie post-opératoire de 6 cures de VAI (Vincristine, Actinomycine, Ifosfamide) a été instaurée. Une rémission clinique et radiologique était notée avec un recul d'un an.

**Figure 1:**
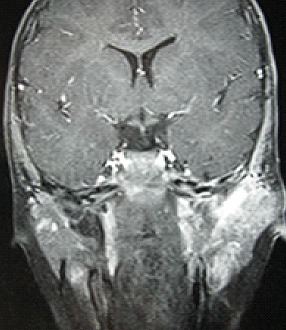
IRM des rochers en coupe coronale: lésion tissulaire du conduit auditif externe gauche, en hyposignal T1 se rehaussant après injection de gadolinium, étendue à l'oreille moyenne et la parotide homotérale.

